# The role of combined FDG and SST PET/CT in neuroendocrine tumors

**DOI:** 10.1111/jne.13474

**Published:** 2024-11-22

**Authors:** Martina Di Franco, Emilia Fortunati, Lucia Zanoni, Stefano Fanti, Valentina Ambrosini

**Affiliations:** ^1^ Nuclear Medicine Alma Mater Studiorum, University of Bologna Bologna Italy; ^2^ Nuclear Medicine IRCCS, Azienda Ospedaliero‐Universitaria di Bologna Bologna Italy

**Keywords:** FDG, NET, neuroendocrine tumors, PET

## Abstract

Somatostatin receptor (SST) positron emission tomography with computed tomography (PET/CT) is the gold standard for functional imaging of neuroendocrine tumors (NETs), but FDG PET/CT is increasingly recognized for its prognostic value, particularly for higher‐grade NETs and to detect disease heterogeneity. Despite the established role of pathological grading, clinical heterogeneity within the tumor burden often complicates accurate prognostication. Evidence suggests FDG PET/CT can outperform WHO grading in predicting outcomes by identifying aggressive, undifferentiated tumor clones that influence long‐term prognosis and treatment decisions. Several grading systems integrating both SST and FDG PET/CT have been proposed to better capture tumor heterogeneity and guide clinical management. Studies demonstrate that FDG PET/CT can influence management in a significant subset of patients, although variably reported. Its use remains variable across centers, also affected by different reimbursement policies and local clinical practices. This review explores the indications to FDG PET/CT in NET and the clinical impact of combined SST and FDG PET/CT imaging.

## 1 INTRODUCTION

Neuroendocrine tumors (NETs) are relatively rare malignancies that originate from the neoplastic transformation of neuroendocrine cells, mostly occurring in the gastro‐entero‐pancreatic (GEP) system and in the lungs, among many reported primary sites.^1^, ^2^ Neuroendocrine Neoplasms (NEN) include epithelial well differentiated NET, poorly differentiated neuroendocrine carcinomas (NEC) and NET of neural type (pheocromocytomas/paragangliomas).^1^ NET are subdivided into three categories according to their proliferation index: G1 (Ki‐67 <3%), G2 (Ki‐67 3%–20%) and G3 (Ki‐67 >20%), with a worsening prognosis at higher grades.^1^

Due to NET typical overexpression of somatostatin receptors (SST), these tumors can be targeted by DOTA peptides‐conjugated radiopharmaceuticals for positron emission tomography with computed tomography (PET/CT) (e.g., [^68^Ga‐DOTA0‐Tyr3]octreotate‐[^68^Ga]Ga‐DOTATATE‐, [^68^Ga‐DOTA0‐Tyr3]octreotide‐[^68^Ga]Ga‐DOTATOC‐, and [^68^Ga‐DOTA0‐1NaI3]octreotide‐[^68^Ga]Ga‐DOTANOC‐) and for therapy with [^177^Lu][Lu‐DOTA0‐Tyr3]octreotate ([^177^Lu]Lu‐DOTATATE).^3^, ^4^, ^5^

SST PET/CT is currently the gold standard for NET functional imaging, being recommended by the SNMMI/EANM guidelines for initial staging at diagnosis, localization of the primary tumor, staging before surgery, selection of patients for PRRT (peptide receptor radionuclide therapy), and 9–12 months post‐PRRT as a new baseline for subsequent treatment planning, as recommended by current EANM/SNMMI guidelines.^3^, ^4^ However, many centers' local practice includes performing SST PET/CT 3 months after the completion of the PRRT course, although aware of the potential risk of false positive findings. Appropriate use criteria also include the indication for patients with suspected NET (in particular in cases of lesions not amenable to biopsy).^6^

[^18^F]fluorodeoxyglucose, [^18^F]FDG (FDG) is the primary and most extensively utilized radiopharmaceutical for PET imaging in oncology. The ENETS and ESMO guidelines include optional FDG PET/CT to study patients with Ki‐67 >10% G2, G3 NET, and NEC,^7^, ^8^ concordant with the well‐known increase in glycolytic activity with rising cellular proliferation index.^9^, ^10^, ^11^

Although the choice of the most appropriate PET radiopharmaceutical is initially guided by pathological grading, clinical evidence indicates a wide heterogeneity in NET in terms of grading within the whole tumor burden, clinical presentation (e.g., functioning vs. non‐functioning; pancreatic primary vs. midgut primary; etc.) and outcome (with survival ranging from weeks to decades depending on grade, pathological features, primary tumor site, and stage at diagnosis). Therefore, pathological grading alone cannot always fully picture the disease behavior, which can be influenced by different cellular clones at different levels of proliferation and differentiation. These clones can co‐exist at the time of the diagnosis (intra‐patient or intra‐lesion heterogeneity) or be selected afterwards during the natural history of the disease or during treatment.^12^, ^13^, ^14^

The use of FDG to complement SST PET/CT has been suggested to fully characterize the whole tumor burden biology/heterogeneity by both demonstrating the SST‐expressing tumor component and by excluding the presence of FDG‐positive undifferentiated tumor clones that may ultimately drive the long‐term prognosis. The mere detection of SST‐negative findings on diagnostic CT is not sufficient to provide predictive and prognostic data that can be obtained by FDG‐metabolic assessment.

Although the double‐tracer approach has been suggested for decades, FDG PET/CT is still variably performed across different centers and countries (depending on reimbursement policies, different procedure availability, local diagnostic protocols, and local expertise) and standardized criteria for its use are lacking. The evidence that most NET patients will show a slow growing/indolent disease, together with different national reimbursement policies, are the most cited arguments against the wide spread use of FDG in this clinical setting. Treatment strategies for advanced forms include surgery, somatostatin cold analogues therapy, PRRT with [^177^Lu]Lu‐DOTATATE, chemotherapy or other systemic treatments.^8^ When positive, FDG PET/CT can influence the subsequent treatment decision and follow‐up (e.g. if spatially mis‐matched lesions are detected, PRRT is generally contraindicated).

Aim of the present manuscript is to review the current knowledge on the combined use of FDG and SST PET/CT imaging, focusing on its relevance on the clinical impact.

## 2 PROGNOSTIC ROLE OF FDG PET/CT IN NENs VERSUS HISTOLOGICAL GRADE

Since NET prognosis is widely variable, accurate prognostication is vital to guide the optimal disease management. NET risk stratification is traditionally based on the WHO grading system. However, histopathological analysis cannot always depict the whole tumor burden heterogeneity (e.g., pathological sampling is often performed in the easiest‐to‐reach lesion, that may not necessarily be the most aggressive one) and may change over the course of the disease natural course. This issue was outlined by Singh et al., who reviewed the analysis of multiple specimens obtained from the same 43 NET patients during the course of the disease, hypothesizing that Ki‐67 index may change (from primary to metastasis, from primary to recurrence or during progression). More than 1/3 of the repeated biopsies (39 cases of the primary NET and a metastatic focus; 4 cases with specimens from multiple metastatic foci) resulted in a different WHO grade compared with the first pathological assessment. In most cases (12/16) re‐biopsy showed disease upstaging: 5 (31%) passed from G1 to G2, 2 (13%) from G2 to G3, and 5 (31%) from G1 to G3.^15^

Whole tumor burden heterogeneity was also reported by Shi et al. in a much larger cohort of 779 patients with confirmed GEP‐NEN. In 35/779, samples from both the primary and metastatic sites were available and Ki‐67 variability was observed in 54.3%, with a change in WHO grade occurring in 8.6% (*n* = 3/35: 1 passed from G1 to G2, 1 from G1 to G3, and 1 from G2 to G1). The cases with intra‐tumor heterogeneity showed a poorer prognosis in terms of OS (hazard ratio 6.800, 95% confidence interval [CI], 1.833–25.230; *p* = .0346).^16^

Potential prognostic factors were studied by several authors^11^, ^17^, ^18^, ^19^ (Table 1). Binderup et al. prospectively analyzed various tumor's characteristics among which WHO grading and FDG PET/CT positivity in 166 individuals with proven GEP‐NET, observed over a median 10‐year follow up period. The authors found a strong and significant association between FDG PET/CT positivity (*n* = 90/166) and both PFS (HR = 2.5; *p* < .001) and OS (HR = 3.8; *p* < .001). The result was confirmed also after excluding G3 tumors (median PFS for FDG‐positive vs. FDG‐negative = 2 vs. 4.8 years; median OS for FDG positive vs. FDG‐negative = 4.1 vs. not reached). Conversely, when analyzing the prognostic significance of 2010 WHO grading, no significant differences were found in terms of OS between G1 and G2 (*n* = 153 patients) and, for this subgroup, FDG positivity was the only identifier of high risk of death (hazard ratio: 3.6; 95% CI, 2.2–5.9; *p* = .001).^17^

**TABLE 1:** Key papers main findings.

Author, journal, year	Study design	No. of patients	Primary site	WHO Grade	Indication to PET	FDGpos:FDGneg	mPFS (mo)	mOS (mo)
Chan et al.^11^ Theranostics 2017	Single center retrospective study	62	GEP 79% Lung 8% Unknown 5% Thymus 5% Breast 3%	G1: 23% G2: 53% G3: 19% Unknown 5%	Not specified	51:11	—	P1–4: not reached; P5: 11
Binderup et al.^17^ JNM 2021	Single center prospective study	98	GEP 90%; Other and Unknown primary 10%	G1: 48% G2: 46% G3: 1%	Not specified. Consecutive recruiting	90:76	FDGpos: 1.8 years FDGneg: 4.8 years	FDGpos: 3 years FDGneg: Not reached
Karfis et al.^19^ Oncotarget, 2020	Single center retrospective study	85	GEP	G1: 24.7% G2: 54.1% G3: 21.2%	Not specified	57:28	All: 12.9 C1: 40.1 C2: 11.9 C3: 7	All: 40.1 C1: 103.2 C2: 35.3 C3: 14.0
Langen Stokmo et al.^20^ J Neuroendocrinology, 2022	Multi center retrospective study	66	GEP or CUP with predominance of GI metastases	G3: 21% NEC: 79%	NS	66 FDGpos	—	Low tMTV 21.2 High tMTV 5.7 Low tTLG‐22.8 High tTLG 5.7
Ezzidin et al.^21^ JNM 2014	Dual‐center retrospective study	89	GEP	G1: 20% G2: 58% G3: 22%	Staging	79:9 (mG1 T/L < 1)	—	mG1: not reached mG2: 55 mG3: 13
Rinzivillo et al.^22^ The oncologist 2018	Dual‐center retrospective study	93	GEP	G1: 28% G2: 52% G3: 20%	Ns	62:31	FDGpos: 9; FDGneg: 50	FDGpos: 60; FDGneg: not reached
Bahri et al.^23^ JNM 2014	Single‐center prospective study	38	GEP	Ki‐67 < 2%: 34% Ki‐67 2–15: 37% Ki‐67 >15: 18% Na: 11%	Staging	15:23	FDGpos: 3 FDGneg: 71	FDGneg: 119.5 FDGpos: 15
Zhang et al.^24^ JNM 2020	Single center retrospective study	495	GEP 73.9%, Lung 7.7%, CUP 9.9%, Others 8.5%	G1: 23.6%, G2: 49.5% G3: 5.9% Na: 21%	Pre‐PRRT assessment	382:113	FDGpos: 18.5 FDGneg: 24.1	FDGpos 53.2 FDGneg 83.1
Chan et al.^25^ British Journal of Cancer	Multi center retrospective study	319	GEP	G1: 29% G2: 51% G3: 15% Unknown: 5%	Ns	NETPET Score: P1 88% P2–4 28% P5 12%	—	P1: 101.8 P2–4: 46.5 P5: 11.5
Chan et al.^26^ JNM 2024	Multi‐center retrospective study	44	GEP	G1: 5% G2: 48% G3: 48%	Ns	44	—	Using median tumor discordant volume as a cut‐off: Low‐TDV (<43.7 cm^3^): 23.8 High‐TDV (>43.7): 9.4

Few studies evaluated FDG volumetric parameters as prognosticators in NET. Langen Stokmo et al. retrospectively divided a cohort of 66 patients with high grade GEP‐NEN (14 NET G3 and 52 NEC) into low and high metabolic groups, respectively, based on median total metabolic tumor volume (tMTV = 208 cm^3^) and total lesion glycolysis (tTLG = 1899 g). Both parameters were strong prognosticators: median OS in the low versus high tMTV‐group and in the low versus high tTLG‐group were, respectively, 21.2 versus 5.7 months (HR 2.53, *p* = .0007) and 22.8 versus 5.7 months (HR 2.42, *p* = .0012).^20^

A grading system based on FDG‐avidity‐only was proposed by Ezzedin et al., who observed a population of 89 patients with confirmed inoperable metastatic GEP‐NEN staged with complementary FDG PET/CT, during a follow‐up period of 38 months. The authors created a three‐grade system using tumor‐to‐liver SUV ratio (TTL) of a target lesion to define the thresholds: mG1 (TTL = <1), mG2 (TTL = 1–2.3) and mG3 (TTL >2.3). Patients in mG1, mG2, and mG3 were, respectively, 9, 22, and 57. On a multivariate analysis, mG3 status was an independent predictor of survival, with an HR of 4.7 (95% CI, 1.2–7.0).^21^

Rinzivillo et al. aimed to define the role of FDG PET/CT in a population of advanced entero‐pancreatic NET with known progressive disease (68.8%) or stable disease (31.2%) (*n* = 93 patients, 69 pancreatic, and 24 with small intestinal NET). FDG PET/CT was positive in 58/64 patients with progressive disease (PD) and 4/29 of patients with stable disease (SD), being significantly associated with disease progression. FDG PET/CT sensitivity and specificity to detect progression were 90.6% and 86.2%, respectively.^22^

Sato et al. retrospectively analyzed 72 patients with a diagnosis of pancreatic NET who underwent a FDG PET/CT, finding a positive association between FDG positivity, advanced disease, and shorter recurrence‐free survival.^27^

Overall, the prognostic role of FDG is confirmed across all tumor grades, primary site, and treatment,^23^, ^24^, ^28^, ^29^, ^30^ also in prospective cohorts,^31^ and several grading systems were proposed to interpret combined PET/CT imaging.

## 3 GRADING SYSTEMS IN DUAL/COMBINED SST AND FDG PET/CT

**FIGURE 1:**
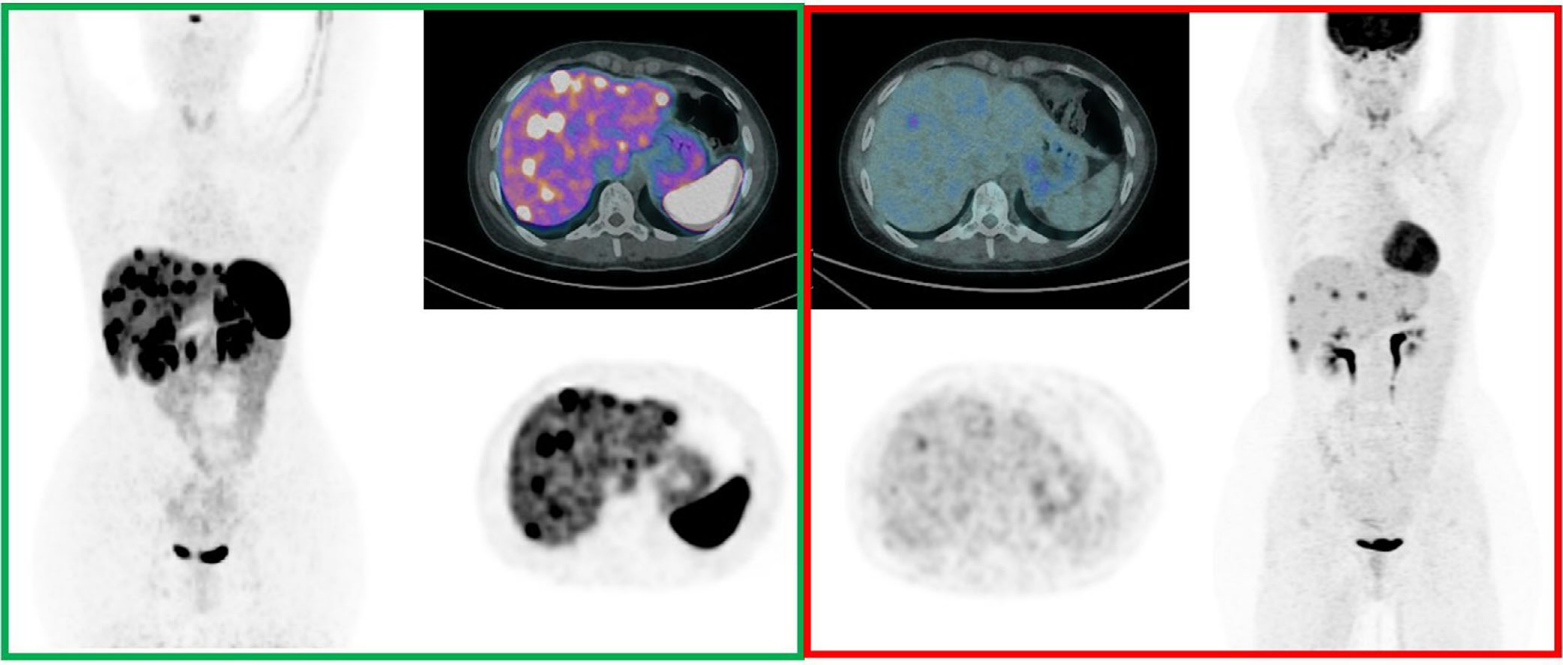
[^68^Ga]Ga‐DOTANOC (green box) and [^18^F]F‐FDG PET/CT (red box) images, acquired 1 day apart, in a 27 years old female patient. Indication was restaging after chemotherapy (CAPTEM) of a pancreatic NET G3 (Ki‐67: 28%). [^68^Ga]Ga‐DOTANOC PET/CT detected multiple focal lesions of liver uptake (green box), only some of them also demonstrated [^18^F]F‐FDG PET/CT uptake (matched findings) (red box).

When performing both SST and FDG PET/CT, the findings can be complex to interpret: the scans can be concordant or discordant in terms of overall positivity/negativity or single lesions diverging uptake (spatially matched/mismatched) (Figure 1).

Since the presence of disease heterogeneity may impact treatment decisions, several grading systems were proposed to interpret combined SST and FDG PET/CT.

The NETPET score was proposed by Chan et al. in 2017 (P0 = negative scan on both tracers; P1 = SST positive/FDG negative lesions only; P2–4 = positivity on both tracers, with gradual escalation in FDG uptake; P5 = presence of FDG‐positive/SST‐negative significant disease)^11^ and later validated through a multi‐institutional analysis (median OS/TTP of 101.8/25.5 months for P1, 46.5/16.7 months for P2–4, and 11.5/6.6 months for P5).^25^

The NETPET score also provides indications of which molecular phenotypes could benefit the most from PRRT (P1–P3 and some of the P4), which are unlikely to respond (P5) and which could be suitable for PRRT with additional treatments (some of the P4, depending on the number of lesions with higher FDG uptake than SST uptake).

Karfis et al. proposed an easy‐to‐use three‐grade scale based on three different clinical scenarios: C1 if all lesions are FDG‐negative and 68Ga‐DOTATATE‐positive; C2 if patients have one or more FDG positive lesions, all of which are 68Ga‐DOTATATE positive (spatially concordant FDG‐pos/SST‐pos); C3 if patients have one or more FDG positive lesions, with at least one being 68Ga‐DOTATATE negative (spatially discordant FDG‐pos/SST‐neg).^19^

Zhou et al. recently evaluated 51 patients with newly diagnosed NET who underwent both SST and FDG scans at staging, stratifying the scans according to three different patterns (A = SST‐positive and FDG‐negative, B = SST‐positive and FDG‐positive, C = SST‐negative and FDG‐positive). FDG‐positivity correlated with patients' outcome (PFS = 10 months in the FDG‐positive/SST‐negative group, 30 months in the concordant FDG positive‐SST positive group and not reached in the FDG‐negative group) at univariate and multivariate analyses (*p* = .026 and .039, respectively), while grade and stage did not. Moreover, FDG detected additional lesions in 9/51 patients, and caused a change of stage in three of them (one from unknown to IV, one from unknown to II, and one from III to IV). Finally, different uptake patterns were observed between primary and metastatic sites (44% of patients with pattern A of the primary had FDG‐positive metastases).^18^

Zidan et al. employed a similar scale in a retrospective cohort of 56 patients with biopsy‐proven Typical carcinoid (TC; 22/56) and Atypical carcinoid (AC; 34/56), studied with both 68Ga‐DOTATATE and FDG (score 1: if all lesions were negative on both tracers, score 2: if all lesions were SST‐positive/FDG‐negative, score 3: if all lesions were SST‐positive but some/all were spatially concordant FDG‐positive, score 4: spatially discordant FDG‐positive and SST‐negative lesions). Again, a high inter‐ and intra‐patient's heterogeneity was observed and only score 2 (FDG‐negative) and 3 (matched SST‐FDG positivity) were considered suitable for PRRT.^32^

Overall, all the above cited scoring systems indicate that a crucial condition to identify is the presence of metabolically active/SST‐non‐expressing disease. These so called “mis‐matched” lesions have been considered indicators of a poorer prognosis and are unanimously deemed to contraindicate PRRT alone. A quantification of this parameter was recently proposed by Chan et al. in a multicentric analysis, that measured the “total discordant volume” (TDV) by summing the volumes of FDG‐positive/SST‐negative mis‐matched lesions among 44 individuals with a GEP‐NET. OS was longer in the low‐TDV cohort than in the high‐TDV cohort (median volume, 43.7 cm^3^; survival time, 23.8 vs. 9.4 months; hazard ratio, 0.466 [95% CI, 0.229–0.948]; *p* = .0221).^26^ The possibility of shifting from the assessment of single lesions' features to the assessment of the whole tumor burden represents a crucial new way of interpreting PET images and a novel approach on which to structure the subsequent patients' management. Although the methods for whole tumor volume segmentation are not fully standardized, they are expected to overcome the limitations represented by both morphological (RECIST) and functional (SUV‐based) criteria previously applied for both disease staging and therapy response assessment.

A limitation of the works conducted so far on double tracer imaging is the retrospective study design and the consequent heterogeneous patient selection for the FDG scan, often based on a clinical need basis. Prospective studies, ideally also including whole tumor volume segmentation among other parameters, are warranted to fully understand the diagnostic and therapeutic impact of patients' stratification based on combined molecular imaging features.

## 4 USE OF FDG PET/CT IN G1 NETs

The “flip–flop” phenomenon has been described as the process of SST loss that can coincide with the rise of glucose metabolism in highly proliferating NET cells.^10^, ^33^ However, unexpected patterns have been observed^34^: SST overexpression is consistently documented in higher grades NET, which are increasingly being considered for PRRT.^35^ Conversely, FDG uptake has also been described in low grade tumors.

A meta‐analysis by Liu et al., encompassing 30 works and 3401 patients, showed pooled sensitivities for G1, G2, and G3 of, respectively, 92.3%, 90.2%, and 57.8% for SST PET/CT and of 37.8%, 55.4%, and 71.2% for FDG PET/CT, demonstrating the frequent presence of FDG uptake also among G1 tumors, albeit limited.^10^ In the above mentioned paper of validation of the NETPET score, FDG‐positivity could be detected across all grades, albeit more frequently in G2–G3 (59% in G1, 73% in G2, and 98% in G3).^25^

Although FDG‐positivity has been reported in G1 NET, the interpretation of the published data is not straightforward. For example, some studies analyzed G1 and G2 cases as a single group^36^ and most often the intercurrent time between pathological grade assessment and detection of FDG‐positivity was not detailed (e.g., pathological assessment may have been performed years before the detection of positivity on the FDG PET, often performed at the time of progression). Binderup et al. reported FDG‐positive foci in 69/140 patients with G1 or G2, without specifying the positivity rate per single grade.^17^ Other authors reported very different rates of FDG‐positivity in G1 in different cohorts: 14/26 patients,^22^ 19/47 patients,^28^ in 7/66 patients,^30^ and in 57% of patients with G1 disease.^18^

Magi et al. retrospectively examined a group of patients with G1 GEP‐NET who underwent FDG PET/CT imaging at baseline (*n* = 38) (e.g., for SST PET negativity or heterogeneous SST PET/CT uptake) or at the time of progression (*n* = 17). Overall, the median time from the histological diagnosis to the FDG PET/CT scan was 5 months. The authors reported FDG positivity in 27/55 (49%) cases: as expected, also in this cohort, FDG positivity in G1 NET was associated with poorer outcome (PFS not reached in FDG PET/CT‐negative patients; PFS = 24 months in FDG‐positive patients, *p* = .04).^37^

The detection of an FDG‐positive tumor component in G1 (and low‐grade G2) NETs, especially if spatially discordant with respect to SSR PET/CT findings, is associated with a more unfavorable outcome. However, upon detection of FDG‐positivity, the change of management is not standardized and should take into account the metabolically active tumor volume. Preliminary evidence supports the role of FDG‐positive discordant tumor volume in clinical decision‐making (as described by Chan et al.^26^) and needs to be validated in larger randomized clinical trials.

Therefore, the issue of whether to perform FDG in G1 needs to be critically evaluated considering the primary site (e.g., pancreatic/lung versus midgut) and the clinical question (taking into account various factors, including, for example, the results of diagnostic CT and SST PET/CT, if eligibility for PRRT is considered, and if there is suspicion of clinical progression). In clinical practice, the indication of FDG PET/CT in patients with NET G1 should therefore be limited to selected cases after multidisciplinary discussion.

## 5 CURRENT INDICATIONS TO FDG PET/CT

In general, the indication to additional FDG‐imaging is discussed at a multidisciplinary level and is mostly driven by the available pathological grade (especially when recently assessed) and follows the comparison of diagnostic CT findings and corresponding SST PET/CT patterns of uptake. FDG PET is also commonly performed in case of rapid clinical deterioration.

However, results from various meetings and surveys among experts indicate distinct scenarios in which clinicians would consider adding an FDG PET/CT. For what concerns higher grade NET, in a recently published consensus document, full agreement (82%) between the participants was reached regarding the use of FDG PET/CT in G3 NET if curative surgery is considered. Moreover, experts agreed to perform additional FDG imaging, irrespective of grade, in all cases presenting SST‐negative lesions evident on the corresponding diagnostic CT, especially if PRRT is indicated. In this survey, consensus was not reached regarding the indication to FDG imaging in case of suspected clinical or radiological progression.^38^ Finally, although not reaching consensus, more than half of the respondents (68%) would biopsy a new FDG PET/CT‐positive lesion, on the background of a prior G1‐2 NET, to re‐assess tumor grade before a change in therapeutic management.^38^ Of course, in this clinical setting, it should be remembered that an FDG positive lesion may also harbor a non‐NET tumor.

The document produced by the EANM Focus 3 multidisciplinary conference reported that 73.9% of the respondents agreed that FDG can be considered for restaging in selected patients (based on grade, and CT/MRI abnormalities that are SST‐negative), if positive at baseline or in cases of rapid progression.^39^ Consensus was also reached on employing FDG in G3 NET and in G1–2 NET with mis‐matched lesions detected at CT and SST‐negative^39^ (Table 2).

**TABLE 2:** Indications for FDG PET/CT imaging based on experts consensus (A: Full consensus; B = high level of agreement).

Grade	Scenario	Level of recommendation	References
Regardless of grade	Diagnostic CT‐positive/SST PET/CT negative findings (staging, restaging, and prior PRRT)	A	[38, 39]
	Suspected Clinical or Radiological Progression	A	[39]
	Restaging if FDG‐positive at baseline	A	[39]
G1–G2	Before re‐biopsy before a change in management	B	[38]
NEC and G3	Staging before curative surgery	A	[38, 39]
	Baseline for chemotherapy initiation	A	[39]

## 6 IMPACT OF FDG PET/CT ON CLINICAL MANAGEMENT OF NENs

Upon detection of FDG positivity, subsequent clinical management changes (in terms of both treatment and follow‐up schedule) may be different among centers (e.g., in particular, when only a single FDG faintly positive mis‐matched lesion is detected).

Assessing the effective clinical impact of FDG results on management is a challenging issue since published studies were mostly retrospective and analyzed cohorts of often selected patients (e.g., in which FDG was indicated following a single case‐based discussion).

Panangiotidis et al. observed that among a cohort of 104 NET patients of all grades (G1 = 36, G2 = 40, G3 = 28; primary site: 16 lung, 33 CUP, 50 GEP, 4 ovary), management was changed based on FDG in only 22/104 (22%) of cases studied with double tracer imaging.^40^

In a cohort of well‐differentiated NET (63 patients included in the final analysis; 60/63 SST‐positive), FDG was positive in 39.7% (25 patients), weakly positive in 17.4% (11 patients) and negative in 42.9% (27 patients). The authors reported that, based on FDG PET findings, a management change (defined as the addition of chemotherapy) was performed in 11 (17%) patients: in 9.6% (3 pts) of G1 and 25% (8 pts) of G2 patients.^41^

Choudhury et al. suggested to perform FDG PET in NET with Ki‐67 ≤5%, in case of treatment naive patients with discordant histopathology/cytology reports, mismatched lesions (a setting in which FDG is likely also to change management), for restaging if progression is suspected or in patients unresponsive to somatostatin analogs or PRRT treatment. The authors suggest that, on the other side, in treatment naïve cases, with resectable SST‐positive disease without distant metastasis on CT, FDG PET/CT is unlikely to be helpful and can be omitted.^41^

With respect to G1 NET, a change in clinical management was reported by Magi et al. in 52.7% of patients after the FDG PET/CT scan, either when negative (leading to somatostatin analogs, active surveillance or PRRT) or positive (resulting in choosing everolimus or PRRT).^37^ By contrast, in the above mentioned paper by Panagiotidis et al. a change in the therapeutic strategy was reported in only 1/36 G1 NET patients based on FDG alone and in 11/36 patients based on the SST‐FDG combination.^40^

However, in the study by Partelli et al. including 49 consecutive pancreatic NET patients (of all grades), the authors concluded that therapy was not influenced by the performance of combined SST and FDG PET/CT, and suggested a potential higher impact in the setting of pancreatic NET with Ki‐67 >10%.^36^

## 7 USE OF FDG PET/CT BEFORE PRRT

The fundamental prerequisite for PRRT eligibility is the performance of SST PET/CT imaging to demonstrate sufficient SST‐uptake in all lesions at the baseline evaluation, adhering to the principle of “what you see is what you treat.” Accordingly, lesions that are detected on CT or FDG PET/CT (with significant FDG uptake, above normal liver parenchyma) but not on SST PET/CT, known as spatially mis‐matched lesions, generally contraindicate PRRT.

However, FDG imaging is currently not part of the routine pre‐PRRT protocol in most centers. In a recently published study based on the Spanish national registry^42^ of 522 patients treated with at least one cycle of PRRT, most patients (73%) were not studied with FDG before PRRT. When performed, FDG resulted discordant with SST imaging in 17% of cases, not concordant in 9.6%. When evaluating the need to perform FDG PET/CT before PRRT, several issues need to be debated. The first question is whether the detection of FDG uptake in SST‐positive lesions should discourage PRRT. Another issue is whether the additional information obtained by FDG would lead to a change in therapeutic planning, given the new proposals of combination therapies, retreatments or personalized schemes.

Several studies indicated that a positive FDG is associated with a worse outcome after PRRT: in a meta‐analysis including 12 studies and 1492 patients, FDG‐negative patients before PRRT had a higher disease control rate and longer PFS and OS as compared with FDG‐positive cases.^43^

Rodrigues et al. also evaluated the value of FDG in 40 patients who received a second course of PRRT (re‐treatment): a more favorable outcome was observed in case of a FDG‐negative scan after the second course (*n* = 26; median OS = 145.5 months; 95% CI, 83.34–207.67) as compared with FDG‐positivity (*n* = 14; median OS = 95.06 months; 95% CI, 48.36–141.77).^44^

Other studies aimed to investigate FDG PET positivity as a predictive factor for PRRT response, mostly finding early disease progression rates in patients with matched SST‐ and FDG‐positive lesions receiving PRRT.^29^, ^45^

FDG is prognostic also in patients undergoing PRRT. Zhang et al. retrospectively analyzed a large population of patients treated with PRRT (*n* = 495; cancer of unknown primary = 49, lung = 38, midgut = 139, others = 42, pancreas = 199, rectum = 20, stomach = 8; G1 = 117, G2 = 245, G3 = 29, not assessed = 104), identifying FDG PET positivity as an independent negative prognostic factor, being associated with shorter median PFS (18.5 vs. 24.1 months) and OS (53.2 vs. 83.1 months).^24^ In the work by Binderup et al., among all patients receiving PRRT, FDG‐negative cases had a longer survival than FDG‐positive patients. However, among all FDG‐positive cases, survival was longer if patients had received PRRT compared with FDG‐positive cases not receiving PRRT.^17^ These results suggest that, although FDG positivity remains an indicator of a poorer prognosis, patients with an FDG‐positive scan can still benefit from PRRT.

The NETPET score identified, among the FDG‐positive patterns, the ones that are likely or unlikely to benefit from PRRT. The latter coincides with the presence of (P4b and P5 patterns).^11^

With respect to the FDG‐based personalized approach to PRRT, some authors hypothesized a possible treatment intensification with chemotherapy in cases of FDG positivity, for the purpose of targeting both well differentiated and more aggressive clones. Kashyap et al. retrospectively analyzed those patients (*n* = 52) who received PRRT in combination with radiosensitizing 5‐fluorouracil. The patients had FDG‐avidity, and/or other adverse prognostic factors. After a median follow‐up period of 36 months, the outcome was favorable, with a median OS not achieved and a median PFS of 48 months. When response assessment was possible, 30% of patients demonstrated a complete or partial response and 68% achieved partial stability 3 months after the treatment.^46^

Similarly, the combination between chemotherapy and PRRT was also investigated by Parghane et al. in 38 patients with non‐mismatched FDG‐positive lesions (estimated PFS rate of 72.5% and OS rate of 80.4% at 36 months^47^) and by Nicolini et al. in 37 individuals with FDG‐positive G1, G2, and G3 NET (median PFS of 31.4 months [17.6–45.4], and median OS not reached).^48^

In conclusion, since FDG before PRRT is not standard practice and it is variably performed, subsequent decision to perform PRRT should follow multidisciplinary discussion, taking into account not only the discordant tumor volume, but also patient‐dependent parameters, for example, performance status, kidney function, age, and life expectancy, especially when considering chemotherapy as an alternative or concomitant treatment. On the contrary, the presence of matched lesions is not a contraindication to PRRT, although a lower response rate is expected.

## 8 CONCLUSION

Combined SST and FDG PET/CT allows the assessment of disease heterogeneity on the whole tumor burden, providing crucial information on disease heterogeneity and prognosis. However, FDG is still variably performed in NET in different centers. According to expert consensus, FDG PET is particularly helpful in cases of diagnostic CT‐positive lesions resulting negative on SST PET/CT, regardless of tumor grade, especially when PRRT is considered. FDG is also useful in NET G3 before surgery and for restaging, especially if progression is suspected. Upon detection of FDG‐positivity, clinical management is variably changed. To fully estimate the impact of additional FDG imaging, prospective well‐designed studies are needed.

## AUTHOR CONTRIBUTIONS

**Martina Di Franco:** Writing – review and editing; writing – original draft. **Emilia Fortunati:** Writing – review and editing. **Lucia Zanoni:** Writing – review and editing. **Stefano Fanti:** Conceptualization; writing – review and editing. **Valentina Ambrosini:** Conceptualization; writing – review and editing; supervision.

## CONFLICT OF INTEREST STATEMENT

Martina Di Franco and Emilia Fortunati declare no conflicts of interest. Valentina Ambrosini reports personal fees from EANM, ESMIT, ESMO, Elma Academy, AAA, and Cineca outside the submitted work. Stefano Fanti reports, outside the submitted work, personal honoraria from Novartis and personal fees from AAA, Amgen, Astellas, Bayer, Debio, GE Healthcare, Immedica, Janssen, Sofie, and Telix. Lucia Zanoni reports, outside the submitted work, personal fees from Springer (as a book editor).

### PEER REVIEW

The peer review history for this article is available at https://www.webofscience.com/api/gateway/wos/peer-review/10.1111/jne.13474.

## Data Availability

Data sharing not applicable to this article as no datasets were generated or analysed during the current study.
